# Brain Vital Signs in Elite Ice Hockey: Towards Characterizing Objective and Specific Neurophysiological Reference Values for Concussion Management

**DOI:** 10.3389/fnins.2021.670563

**Published:** 2021-08-05

**Authors:** Frederick R. Carrick, Guido Pagnacco, Sergio F. Azzolino, Melissa Hunfalvay, Elena Oggero, Tory Frizzell, Christopher J. Smith, Gabriela Pawlowski, Natasha K. J. Campbell, Shaun D. Fickling, Bimal Lakhani, Ryan C. N. D’Arcy

**Affiliations:** ^1^University of Central Florida College of Medicine, Orlando, FL, United States; ^2^MGH Institute of Health Professions, Boston, MA, United States; ^3^Centre for Mental Health Research, University of Cambridge, Cambridge, United Kingdom; ^4^Centre for Mental Health Research in Association with University of Cambridge, Cambridge, United Kingdom; ^5^Department of Electrical and Computer Engineering, University of Wyoming, Laramie, WY, United States; ^6^BrainNET, Health and Technology District, Vancouver, BC, Canada; ^7^Centre for Neurology Studies, HealthTech Connex, Vancouver, BC, Canada; ^8^DM Centre for Brain Health, Department of Radiology, University of British Columbia, Vancouver, BC, Canada

**Keywords:** electroencephalography (EEG), event-related potentials, brain vital signs, ice hockey, normative data

## Abstract

**Background:** Prior concussion studies have shown that objective neurophysiological measures are sensitive to detecting concussive and subconcussive impairments in youth ice-hockey. These studies monitored brain vital signs at rink-side using a within-subjects design to demonstrate significant changes from pre-season baseline scans. However, practical clinical implementation must overcome inherent challenges related to any dependence on a baseline. This requires establishing the start of normative reference data sets.

**Methods:** The current study collected specific reference data for *N* = 58 elite, youth, male ice-hockey players and compared these with a general reference dataset from *N* = 135 of males and females across the lifespan. The elite hockey players were recruited to a select training camp through CAA Hockey, a management agency for players drafted to leagues such as the National Hockey League (NHL). The statistical analysis included a test-retest comparison to establish reliability, and a multivariate analysis of covariance to evaluate differences in brain vital signs between groups with age as a covariate.

**Findings:** Test-retest assessments for brain vital signs evoked potentials showed moderate-to-good reliability (Cronbach’s Alpha > 0.7, Intraclass correlation coefficient > 0.5) in five out of six measures. The multivariate analysis of covariance showed no overall effect for group (*p* = 0.105), and a significant effect of age as a covariate was observed (*p* < 0.001). Adjusting for the effect of age, a significant difference was observed in the measure of N100 latency (*p* = 0.022) between elite hockey players and the heterogeneous control group.

**Interpretation:** The findings support the concept that normative physiological data can be used in brain vital signs evaluation in athletes, and should additionally be stratified for age, skill level, and experience. These can be combined with general norms and/or individual baseline assessments where appropriate and/or possible. The current results allow for brain vital sign evaluation independent of baseline assessment, therefore enabling objective neurophysiological evaluation of concussion management and cognitive performance optimization in ice-hockey.

## Introduction

### Background

Individuals participating in contact sports are at higher risk for concussion and traumatic brain injury (McCrory et al., 2017). The current standard for concussion assessment in the literature is symptom monitoring, with only a small minority of research studies using additional outcome measures (Haider et al., 2018). Concussions can result in very subtle neurological effects that might not be detected as visible symptoms by either the individual or clinicians. In fact, it has been shown that neurological deficits in areas such as attention exist well after observable symptoms have been resolved (Gosselin et al., 2006; Fickling et al., 2019b). Subjective symptom assessment remains important and can play a major role in treatment programs such as those that incorporate exercise as medicine (Leddy et al., 2018). However, there is an increasing movement to combine these assessments with objective, neurophysiological measures of concussion in order to better quantify injury severity and monitor change over time (Smith et al., 2017; Pender et al., 2020). Recently, studies have demonstrated the ability of rapid objective assessments based on electroencephalography (EEG) that show high sensitivity and specificity to detecting concussion and concussion-related neurological changes (Boshra et al., 2019; Fickling et al., 2019b; Bazarian et al., 2021).

Event related potentials (ERPs) are small stimulus time-locked responses that can be extracted from EEG through signal averaging to obtain a specific record of sensory, perceptual, and cognitive brain activity (Patel and Azzam, 2005; Ghosh Hajra et al., 2016). ERPs are identified by distinct waveforms that represent the polarity, latency and amplitude of brain responses to the reception and processing of stimuli. ERPs have been historically limited to experimental laboratories due to lengthy test times and non-standardized procedures. To facilitate the clinical translation of ERPs, the brain vital signs framework compressed and standardized the ERP procedure into a rapid and automated 6-min evaluation of three well established auditory event-related potential (ERP) responses, the N100, P300, and N400 Ghosh Hajra et al. (2016).

The brain vital sign frameworks uses a combination of auditory tone and spoken word stimuli to elicit the N100, P300, and N400 ERPs, as responses along the continuum of information processing from low-level sensory to higher level cognitive processing. Auditory tones with random embedded deviants elict the N100 and P300. The N100, first demonstrated in 1939, represents the brain’s sensory response to hearing tones and is indexed as a measure of auditory sensation (Davis, 1939; Näätänen and Picton, 1987). The P300 is derived from the brain’s response to random changes of deviant tones within the sequence. It is one of the most extensively researched ERPs across a wide range of brain conditions, it is considered to represent the cognitive processes of attention and memory as well as consciously-maintained working memory processing (Sutton et al., 1965, 1967; Polich, 2007). Finally, spoken word pairs elicit the N400 ERP. Half of the word pairs are semantically congruent (i.e., bread-butter) and half are incongruent (bread-window). The N400 then represents an index of cognitive processing in language, as the brain reacts to the unexpectedness of the semantic incongruency (Kutas and Hillyard, 1980; Ghosh Hajra et al., 2018). The N400 is most affected by language comprehension factors and has been studied in associated neurological conditions such as learning disorders, stroke, TBI, dementia (D’Arcy et al., 2003; Taylor and Olichney, 2007; Schulz et al., 2008; Steppacher et al., 2013; Abel et al., 2018).

Each of these ERP peaks are evaluated for their amplitude, representing the magnitude of simultaneous neural recruitment related to the stimulus, and the latency, representing the speed of response to the stimulus. The spectrum of cognitive processes evaluated (i.e., sensation, attention, and cognitive processing) enable these measurements to be sensitive to subtle neurological impairments in information processing that occur following a sports concussion (Fickling et al., 2019b). Technologically, the collection of brain vital signs with a standardized system at the point-of-care requires a specific device implementation (Ghosh Hajra et al., 2016). The NeuroCatch Platform (NeuroCatch^®^ Inc., Surrey, BC, Canada) is a Health Canada approved class 2 medical device that provides portable, rapid and automated acquisition, display, analysis, storage, reporting, and management of EEG/ERPs in brain vital sign monitoring (Ghosh Hajra et al., 2016). Using this approach, recent brain vital signs research in ice-hockey concussion studies has demonstrated increased sensitivity to both concussive and sub-concussive impacts (Fickling et al., 2019b, 2021).

The prior concussion studies used a within-subjects repeated measures model, where each player acts as their own control (Fickling et al., 2019b, 2021). A baseline test was performed before the start of the season, when a player was theoretically “concussion-free.” However, the challenge with baseline testing is that the assessments must be performed prospectively and can therefore be impractical (Schmidt et al., 2012). While possible in research, evaluation in clinical practice is significantly improved with specific neurophysiological reference data. With specific reference data in addition to more comprehensive general normative datasets, it is then possible to better interpret individual results with respect to statistical frameworks along with the relative effects of demographic variables such as age and sex (Gordon and Konopka, 2005). In addition, known physiological effects related to factors such as repeat testing can also be normatively controlled to aid in specific individual applications. For instance, in a recent study, Smith et al. (2020) verified a slight reduction in the N400 amplitude effects due to habituation during test-retest, which stabilizes across repeat testing (Smith et al., 2020). Importantly, specific reference ranges can help to address key outstanding questions such as: Does a history of playing hockey impact the current results? Questions like this can be empirically stratified to better characterize the relationship between to healthy and concussed athletes. An examination of the sensitivity of normative vs baseline approaches have shown similar abilities to classify injury status (Schmidt et al., 2012). Given that, the definition of “normative” can range greatly based on the population sampled and these are known to affect EEG data (Johnstone and Gunkelman, 2003; Duncan et al., 2009), it is critical to begin narrowing the specificity of norms in order to translate the clinical utility of evoked potentials as brain vital signs.

A systematic review by Conley et al. (2018) found that most EEG studies investigating concussion examined differences between groups of concussed vs. healthy athletes, rather than before and after suspected concussion (Conley et al., 2018). However, several studies have shown that specific factors, which differ for athletes, may affect their EEG results relative to healthy populations of non-athletes (Del Percio et al., 2007; Eckner et al., 2016; Sato et al., 2020). A key question for the practical implementation of brain vital signs, or any quantitative EEG procedure, is therefore whether specific norms differ significantly from general norms? In the absence of an individualized baseline, one can evaluate this question by acquiring a normative reference database with specific controlled variables and attributes for evaluation against both general norms and repeatability in order establish a possible alternative (Duncan et al., 2009).

### Objectives and Hypotheses

This study characterized neurophysiological brain vital signs extracted from auditory ERPs toward developing specific and general reference norms. The primary hypothesis predicted that the ERP responses would be stable over test-retest in Group A, with the exception of the known short-term N400 habituation effect (described above). This critical step is required to enable future comparison of function after injury. A second exploratory question examined whether there were any differences between Group A specific norms and Group B general norms. While the two groups were expected to fall within the same statistically overlapping range, normative age-related differences were anticipated and therefore the question of possible group differences after controlling for the effect of age was also explored.

## Materials and Methods

### Overview

This study was designed as a cross-sectional observational experiment (ClinicalTrials.gov Identifier: Group A: NCT03975023, Group B: NCT03835962). To address the critical need for normative reference values, we enrolled two participant groups. Group A (specific): elite hockey players in which pre-morbid history and future concussion risk is a highly prominent issue. Elite players were recruited to participate in a training camp through CAA Hockey, a management agency for players drafted to professional leagues such as the National Hockey League (NHL). Brain vital signs were collected twice, with each player assessed 2-days apart. Group B (general): A heterogeneous sample of neurologically healthy individuals ranging of all ages and sexes. Brain vital signs were collected once in this group. For both groups, latency and amplitude values for the N100, P300, and N400 were evaluated and the results are considered with respect to future clinical evaluation of concussion within an individual athlete, in the absence of a baseline evaluation.

### Participants

The institutional review board Advarra approved the studies. Informed, written consent/assent was obtained according to the declaration of Helsinki. Participants over the age of majority provided consent. Participants under the age of majority provided assent, in addition to consent from their parents/guardians. Group A: Male elite ice hockey players (*N* = 58; Age: 16.24 ± 0.76 years) were studied. Fifty-one (Yue et al., 2020) were from North America (Canada: 29, United States: 22) and seven from Europe (Sweden: 3; Switzerland: 1; Finland: 1; Slovakia: 2). Forty-three (Nakata et al., 2010) were fluent English speakers. Players did not report previous concussions. For Group B: 135 neurologically healthy individuals living in Canada ranging in age from 8 to 83 years old (mean 40.62 ± 16.88) were recruited. The group consisted of 67 males (Age = 40.54 ± 17.03) and 68 females (Age = 40.71 ± 16.85). All were fluent in English.

### Data Collection

Group A: Brain vital signs testing was completed in a 10-min test session using the NeuroCatch^®^ Platform (NCP) during an international ice hockey camp for male youths in Los Angeles, CA, United States. Groups of five players were scanned in parallel. Distractions were mitigated by performing the scans in a quiet, closed room with all players in the group facing the same direction. Participants (*n* = 46) were retested using the same procedure 2 days later. Different sets of pseudorandomized word pairs were used for the retest, so that participants did not hear the same arrangement of words twice. The dropout for the repeated scans were due to scheduling challenges during the camp. Group B: Brain vital signs testing for the general reference group was completed using the NeuroCatch^®^ Platform in Surrey, BC, Canada. Data were collected in a comparably controlled setting, using the same methodology, acquisition systems, software, and settings as with Group A. Participants were only tested once.

For both studies: EEG data were recorded at 500 Hz sampling rate from three midline scalp electrodes (Fz, Cz, and Pz) embedded in an elasticized g.Nautilus cap (g.tec medical engineering, Austria). A reference electrode was clipped to the right earlobe and disposable Ag/AgCl electrodes were used for electro-oculagram (EOG) recording from the supra-orbital ridge (above) and outer canthus (beside) of the left eye. g.GAMMAsys electrode gel was applied to each location to ensure conductivity. Skin-electrode impedances were maintained below 30 kΩ at each site. Participants were asked to listen attentively to the auditory stimuli, but no active response was required. To reduce motor and ocular artifacts, participants were instructed to sit upright motionlessly, and maintain visual fixation on a cross positioned at eye-level 2 m away.

### Data Processing

Raw EEG data were bandpass filtered from 0.5 to 20 Hz, corrected for ocular artifacts using an adaptive filter (He et al., 2004) and smoothed for feature selection using an epoch-level wavelet filter (Quiroga and Garcia, 2003). After stimulus segmentation and baseline correction, individual trials were rejected if they contained an amplitude above ±75 μV. Proprietary NCP software then automatically identified the latency (in milliseconds) and amplitude (in microvolts) for the N100, P300, and N400 ERPs based on local max/minima within expected temporal ranges. Data from six participants (10.34%) in Group A were excluded due to poor data quality (>than 25% of ERP epochs rejected). Given that the N400 is a measure of language processing, N400 data were not included from the 15 Group A participants who were not fluent English speakers.

### Outcome Measures and Statistical Analysis

Brain vital signs used for outcome measures included the raw amplitudes and latencies from the six evoked potential measures (3 ERP peaks ^∗^2 peak measures). Standardized brain vital sign scores were then created for Group A by comparing mean brain vital signs data to the distributions from Group B to generate a Z-score which was then linearly mapped to a 0–100 scale, a process described by Fickling et al. (2019b). Based on this approach, larger amplitudes and faster latencies were assigned higher scores; and smaller amplitudes and slower latencies were assigned lower scores (Table 1).

**TABLE 1:** Brain vital sign standardized scoring framework.

	**Amplitudes**	**Latencies**
x > μ + 3 σ	Score = 100	Score=0
μ-3σ < x < μ + 3σ	Score = $\left|\frac{(\text{μ}+3\sigma )-x}{6\sigma }\right|$	Score = $\left|\frac{x-(\text{μ}-3\sigma )}{6\sigma }\right|$
x < μ-3σ	Score = 0	Score=100

Standardization allowed for all six outcome measures to be plotted on equivalent 0–100 scales and relative distributions (given that each amplitude and latency metric has a different range and distribution in the general reference group). Grand-averaged ERP waveforms from all participants were also generated at each time point to visually assess test-retest changes in Group A.

Test-retest evaluation for reliability were completed using Intraclass Correlation Coefficients (Two-way mixed, single measures, absolute agreement) and Cronbach’s Alpha. Descriptive statistics for brain vital signs in both groups were generated for all time points. To compare brain vital signs between the groups, and control for the difference in age, a multivariate analysis of covariance (MANCOVA) was completed with group as the main factor and participant age as the covariate. The MANCOVA was completed for Group A-Time 1 and Group B. Age was modeled as a covariate due to the difference in mean age between the two groups, and the fact that there were insufficient age-matched participants from Group B (Table 2). To be consistent, given that Group B only received one scan at one time point, only the first time point from Group A was included.

**TABLE 2:** Descriptive statistics for all groups and time points.

		**Group A—Time 1**	**Group A—Time 2**	**Group B**
N100 amplitude	*N*	52	46	134
	Mean ± SD	9.48 ± 3.52 μV	8.74 ± 3.39 μV	9.98 ± 4.28 μV
	Median ± IQR	9.03 ± 4.72	8.18 ± 3.88	9.62 ± 6.11
	Range (Min-Max)	15.85 (2.38–18.23)	14.44 (3.82–18.27)	19.77 (0.13–19.9)
N100 latency	*N*	52	46	134
	Mean ± SD	109.46 ± 15.44 ms	110.04 ± 14.47 ms	102.96 ± 11.09 ms
	Median ± IQR	106 ± 18	107 ± 18	102 ± 12
	Range (Min-Max)	70 (80–150)	66 (72–138)	76 (74–150)
P300 amplitude	*N*	52	46	135
	Mean ± SD	9.53 ± 4.32 μV	10.07 ± 5.08 μV	10.14 ± 4.38 μV
	Median ± IQR	8.58 ± 6.88	10.10 ± 6.60	9.56 ± 5.85
	Range (Min-Max)	16.73 (2.99–19.72)	26.66 (2.19–28.85)	24.02 (0.25–24.27)
P300 latency	*N*	52	46	135
	Mean ± SD	279.04 ± 38.78 ms	281.57 ± 44.26 ms	281.57 ± 51.36 ms
	Median ± IQR	282 ± 51	289 ± 62	284 ± 64
	Range (Min-Max)	184 (170–354)	206 (178–384)	286 (162–448)
N400 amplitude	*N*	38	33	135
	Mean ± SD	6.26 ± 2.40 μV	5.10 ± 1.56 μV	4.99 ± 2.28 μV
	Median ± IQR	6.62 ± 2.98	4.96 ± 2.26	4.51 ± 2.60
	Range (Min-Max)	13.79 (0.43–14.22)	6.2 (2.02–8.22)	17.65 (0.94–18.59)
N400 latency	*N*	38	33	135
	Mean ± SD	438.26 ± 58.95 ms	440.36 ± 53.40 ms	456.24 ± 69.18 ms
	Median ± IQR	424 ± 86	442 ± 68	458 ± 100
	Range (Min-Max)	278 (318–596)	250 (330–580)	326 (284–610)

Finally, multiple regression models were used to analyse age-adjusted predictions of amplitude and latency on the N100, P300 and N400. All statistical analyses were completed using SPSS (IBM, NY, United States). Figures were created using Matplotlib package for Python.

## Results

### Descriptive Statistics

**FIGURE 1:**
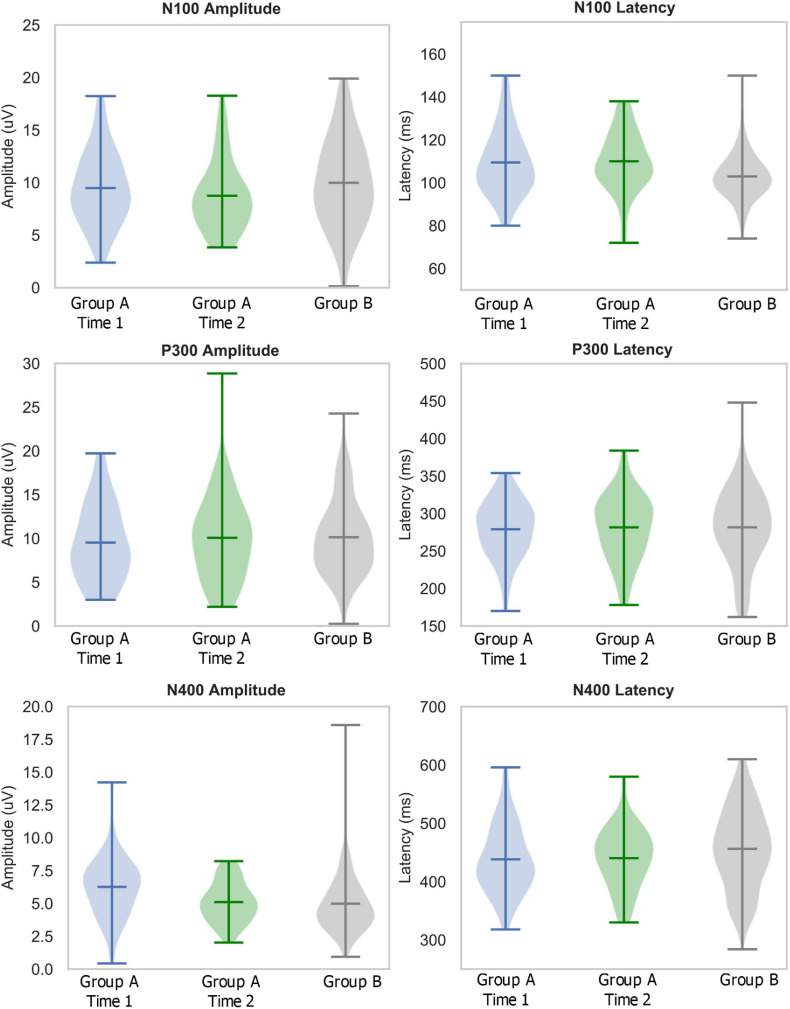
Descriptive statistics: Violin plots describing distributions of relative amplitudes (left) and latencies (right) for N100 (top), P300 (middle), and N400 (bottom) in elite youth ice hockey athletes (Day 1 in blue, Day 2 in green, Ref in Gray). Hashes on the violin plots represent the mean, maximum, and minimum values in the range, with the population probability density shaded. Note: the large ranges in Group A-Time 2 P300 amplitude and Group B N400 amplitude are due to outliers which are included in the violin plots.

Descriptive statistics, including means, standard deviations, medians, interquartile ranges, sample range, minimum and maximum values for each group and time point are shown in Table 1. Table 2 lists the number of participants in different age ranges for each group. To provide an additional visual representation of the relative distributions of the outcome measures, violin plots of peak amplitudes and latencies for N100, P300, and N400 ERPs in both groups and time points are presented in Figure 1. Table 3 outlines the total number of participants in different age ranges for each group.

### Test-Retest Comparison

**FIGURE 2:**
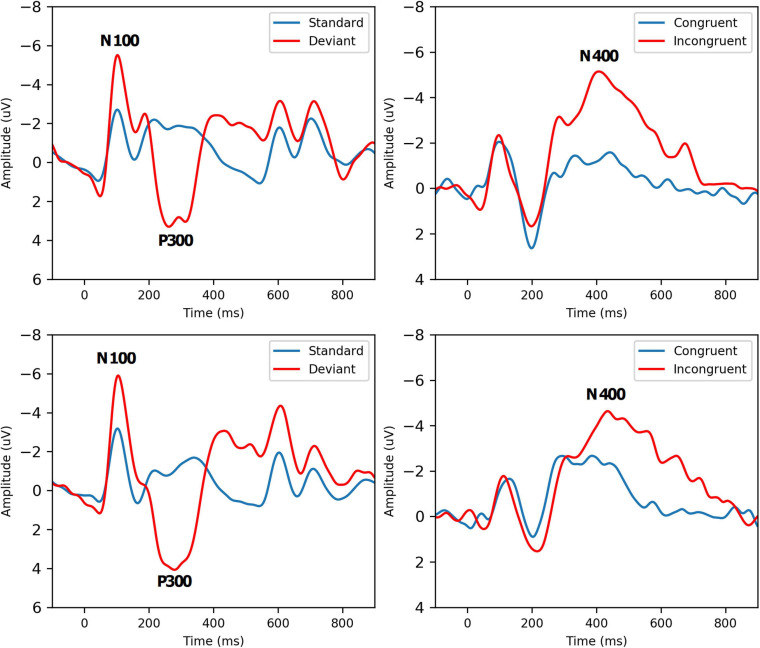
Grand-Average ERPs to standard/deviant tones (left, *N* = 52) and semantic congruent/incongruent word pairs (right, *N* = 38) for Group A Time 1 (Top Row) and Time 2 (Bottom Row). Brain vital sign responses to the pattern changes in different tones (80% standard: low-frequency, low-amplitude, 20% deviant: high-frequency, high-amplitude) generate the well-established N100 and P300 ERP waves. Differences in semantic relationships between congruent (“bread-butter”) and incongruent (“bread-window”) word pairs elicit the N400 negativity. Refer to Ghosh Hajra et al. (2016) for a detailed description of how brain vital signs can be rapidly recorded.

**FIGURE 3:**
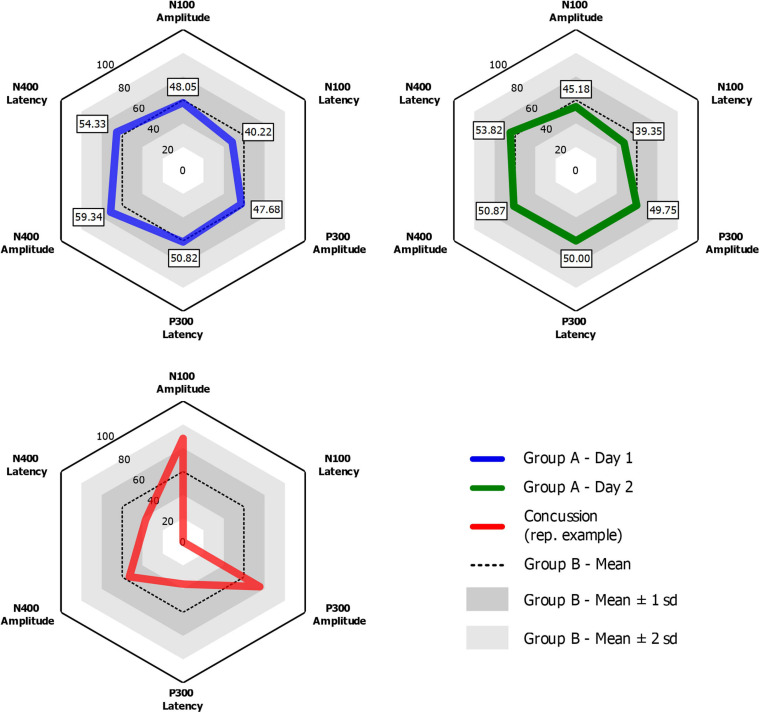
Radar plots for Group A Time 1 (Blue) and Group A Time 2 (Green) showing group-mean elite athlete normalized brain vital signs scores compared to Group B, a general reference database of 135 participants of all ages and sexes. An example radar plot of data from a single concussed ice-hockey athlete for comparison (adapted from Fickling et al., 2019b).

Grand-average ERP waveforms for Group A Time 1 (top row) and Group A Time 2 (bottom row) are presented in Figure 2. Radar plots of group mean elite athlete (Group A) results at each time point, standardized against the general (Group B) reference database are shown in Figure 3. A third radar plot was included as a representation individual concussion (adapted from Fickling et al., 2019b). The results of the statistical test-retest comparisons are represented in Table 4. As expected, there was a significant decrease in N400 amplitude from Time 1 to Time 2 (*F* = 6.061, *p* = 0.019^∗^), but no difference in the other five brain vital signs. In these five measures, the brain vital signs results demonstrated moderate-to-good reliability (Cronbach’s Alpha > 0.7, ICC > 0.5) and showed strong overlap agreement in the waveforms and radar plots.

**TABLE 3:** Total number of participants in different age ranges for each group.

**Age range**	**Group A**	**Group B**
0–10		3
10–20	58	10
20–30		35
30–40		22
40–50		18
50–60		31
60+		16

**TABLE 4:** Statistical analysis for Test-Retest reliability of brain vital signs in Group A.

	**One-way ANOVA**	**Cronbach’s Alpha**	**ICC**	**ICC 95%CI**	**ICC p value**
N100 amplitude (*N* = 46)	*F* = 1.161 *p* = 0.287	0.732	0.577	0.349–0.741	<0.001
N100 latency (*N* = 46)	*F* = 0.033 *p* = 0.857	0.813	0.689	0.499–0.815	<0.001
P300 amplitude (*N* = 46)	*F* = 1.334 *p* = 0.252	0.729	0.572	0.343–0.737	<0.001
P300 latency (*N* = 46)	*F* = 0.397 *p* = 0.532	0.768	0.627	0.414–0.775	<0.001
N400 amplitude (*N* = 33)	*F* = 6.061 *p* = 0.019*	0.503	0.305	−0.008–0.573	0.026
N400 latency (*N* = 33)	*F* = 0.784 *p* = 0.383	0.822	0.699	0.473–0.838	<0.001

*p < 0.05.

### Multivariate Analysis of Covariance

**FIGURE 4:**
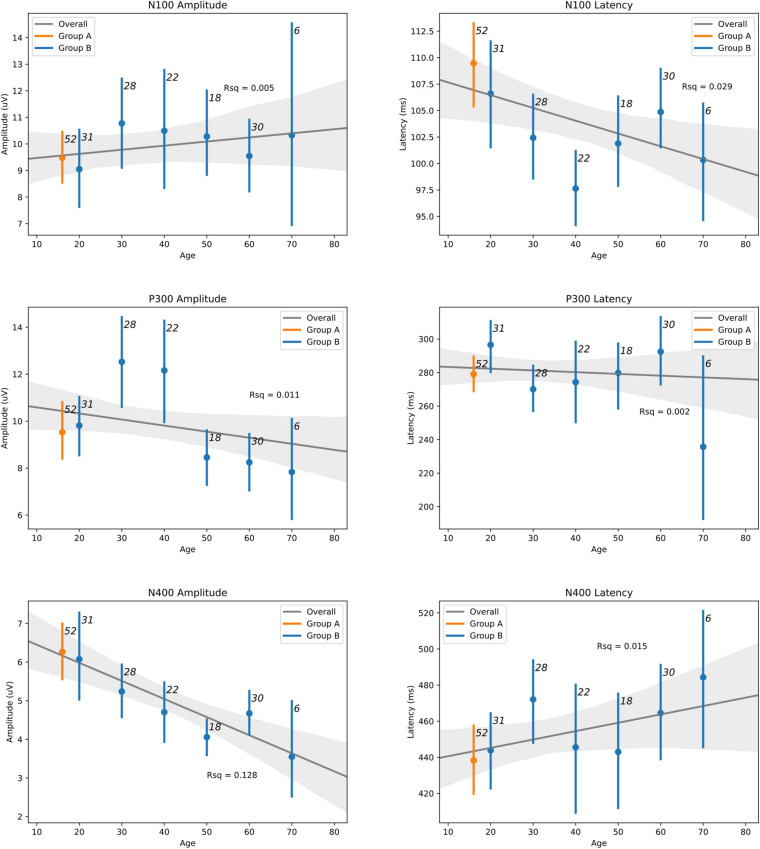
Linear regression models with age-adjusted predictions of ERPs with 95% CIs by binned-age for elite hockey players (Group A—orange) and heterogeneous control group (Group B—blue) groups. Sample size for each bin is indicated adjacent to each CI. Best-fit line is drawn for both groups across the lifespan, with R-squared values also indicated.

Table 5 shows details of the statistical analysis for the MANCOVA comparing Group A-Time 1 and Group B. Effects are shown for multivariate (main effects: all six brain vital signs metrics combined) and univariate (individual brain vital signs) comparisons with *post hoc* covariate-adjusted estimated marginal means. There was no overall significant effect of group in the dataset (*p* = 0.105). As expected, there was a significant overall effect of age as a covariate (*p* < 0.001). Significant univariate effects in N100 latency, P300 amplitude, and N400 amplitude were also present. Differences in P300 amplitude (*p* = 0.014) and N400 amplitude (*p* < 0.001) were explained by age differences, but N100 latency differences remained significant (*p* = 0.022) after adjusting for age. The estimated marginal means showed that the elite athlete group was 6.315 ms slower in N100 latency than the general reference group. Figure 4 displays linear regression models with age-specific predictions of ERPs with 95% CIs by age in 10-year brackets.

**TABLE 5:** Statistical analysis for the multivariate analysis of covariance (MANCOVA) for Group A-Time 1 (time point 1) and Group B.

**Multivariate Tests**															

**Effect**	**Wilk’s Lambda**		**F**			**Hypothesis df**		**Error df**			**Sig**			**Partial Eta Squared**	
Intercept	0.026		1021.110			6.0		164			<0.001*			0.974	
Age	0.855		4.649			6.0		164			<0.001*			0.145	
Group	0.939		1.786			6.0		164			0.105			0.061	

**Tests of Between Subjects Effects**															

	**Corrected Model**				**Intercept**			**Age**				**Group**			
	**F**	**Sig**			**F**		**Sig**	**F**		**Sig**		**F**			**Sig**

N100 amplitude	0.211	0.810			173.89		<0.001***	0.346		0.557		0.01			0.916
N100 latency	5.206	0.006**			2589.27		<0.001***	0.291		0.590		5.36			0.022*
P300 amplitude	3.084	0.048*			245.64		<0.001***	6.153		0.014*		2.27			0.133
P300 latency	0.372	0.690			1186.35		<0.001***	0.728		0.395		0.35			0.558
N400 amplitude	12.768	<0.001***			326.09		<0.001***	15.850		<0.001***		0.09			0.759
N400 latency	1.517	0.222			1436.90		<0.001***	0.758		0.385		0.56			0.455

**Covariate-adjusted estimated marginal means—pairwise comparisons**															

	**Mean difference (B-A)**			**SD error**			**95% CI lower**		**95% CI upper**				**Sig**		

N100 amplitude	−0.099			0.932			−1.938		1.741				0.916		
N100 latency	−6.315			2.728			−11.701		−0.930				0.022*		
P300 amplitude	+1.445			0.958			−0.447		+3.337				0.133		
P300 latency	+6.319			10.757			−14.916		+27.554				0.558		
N400 amplitude	−0.152			0.495			−1.129		+0.825				0.759		
N400 latency	+11.204			14.957			−18.322		+40.731				0.455		

Group is modeled as the main factor and age is modeled as a covariate. Effects are shown for multivariate (main effects: all six brain vital signs metrics combined) and univariate (individual brain vital signs) comparisons with *post hoc* covariate-adjusted estimated marginal means.

*p < 0.05, **p < 0.01, and ***p < 0.001.

*Bold values are where p < 0.05.*

## Discussion

### Main Findings

Hypothesis 1: The specific group of elite ice-hockey players (Group A) were comparable across test-retest (Table 2), except for the expected reduction in the N400. Reliability at the group-level was highly consistent across the group-mean waveforms (Figure 2), with repeatable amplitude and timing in the N100 and P300 and minor reduction in the N400. N400. The well-known N400 habituation effect (Smith et al., 2020) is due to familiarization of word-pair priming in the stimulus sequence when the interval between scans is short. This was still present despite the use of a different arrangement of pseudorandomized word pairs for the second test, suggesting potential habituation to the general concept of congruent vs incongruent pairs instead of (or in addition to) habituation to specific word pairs themselves as previously reported. Of course, some inherent variability in individual test-retest reliability was expected based on daily state (sleep, mood, etc.) and the fact that participants were engaging in contact sport training between the scans. While short term effects have yet to be examined, longer term participation in contact sports has shown to significantly impact brain vital signs in proportion to the number of head impacts received over a specific period (Fickling et al., 2021).

Hypothesis 2: The MANCOVA showed no overall significant difference for group (*p* = 0.105), but a significant overall effect of age as a covariate (*p* < 0.001) was present. At the univariate level, there were significant age-related effects for both P300 amplitude (*p* = 0.014) and N400 amplitude (*p* < 0.001). These are prominent on the regression plots (Figure 4), with a general decrease in both of these components as age increases. While there are some visible overall trends for age in the other four metrics, these were not significant at the multivariate level, suggesting that the multivariate result is largely driven by the significant P300 and N400 amplitude changes. It is also possible that the effect of age on brain vital signs is non-linear. This can be seen in the directional change and subsequent reversal from the 10/20 to 30/40 to 50/60 age groups, particularly in the N100 amplitude, N100 latency, and P300 amplitude (Figure 4). Given that the coefficients of determination at the univariate level (Figure 4) showed that the linear model fit was poor overall, further investigation of age-related changes are required.

After the correction for age, the model found a significant difference only for the N100 latency between the elite and general reference groups. The estimated marginal mean difference between groups for the N100 latency was approximately 6 ms—with N100 peak latency for elite hockey players slightly slower than the general normative reference. Although a small difference, it appears to reflect a specific sample difference. While an underlying explanation of the difference is beyond the scope of the current study, we speculate that sensory modality processing differences may exist for elite sportspeople who may be predisposed toward visual processing compared to auditory processing. Further investigations would need to compare the auditory and visual N100 directly to better explore specific differences in sensory modality processing (Pawlowski et al., 2019).

### Comparisons With Available Data

The results provided a critical first step in the use of brain vital signs evaluation independent of a baseline. They confirm that comparisons are possible through either the specific or general reference value data sets. Given the rapid time advantage of brain vital signs (in minutes), continued development and refinement of normative databases is highly practical. Accordingly, while the current findings provide the initial publication of an open-access database for brain vital signs reference data, continued normative data collection is required.

The distributions of ERP responses were not surprising. The N100, P300, and N400 ERPs have been extensively studied in both healthy and neurological populations given their respective discoveries in 1939 (Davis, 1939), 1967 (Sutton et al., 1967), and 1980 (Kutas and Hillyard, 1980). Detailed systematic reviews and meta-analyses of the N100 (Tomé et al., 2015) and P300 (van Dinteren et al., 2014) literature have provided insight into response features across the lifespan for healthy controls. The regression plots across different age groups corresponded closely with these prior meta-analyses for the N100 and P300.

Interestingly, the current results appear to represent the initial published norms for the N400 response across the lifespan. Given the relationship to higher-level cognitive processing, the N400 is an increasingly important brain vital sign response. Clinical ERP studies have repeatedly demonstrated the critical role of the N400 in acquired brain injury (D’Arcy et al., 2003; Gawryluk et al., 2010) and recently shown the specific importance in understanding subconcussive/subclinical impairment (Fickling et al., 2019b, 2021). The current N400 effects replicated prior empirical characterization of habituation effects. N400 habituation appears to stabilize quickly and show increased sensitivity to subtle cognitive processing improvements even in healthy individuals (Smith et al., 2020). For instance, cognitive training paired with neuromodulation significantly improved cognitive vigilance and reduced the N400 habituation (Smith et al., 2020), suggesting potential application in the optimization of cognitive processing for elite performance (Frehlick et al., 2019).

While preliminary, the specific reference data provide a valuable benchmark for further comparison of elite athlete patterns (Del Percio et al., 2007; Taliep and John, 2014; Sato et al., 2020). For example, elite ice-hockey performance requires speeded sensory-perceptual, attention, and cognitive processing, which can be benchmarked in terms of brain vital signs responses against the norms. An individual athlete can improve athletic performance at the elite level through measuring improved parameters like improving physical performance (Nakata et al., 2010; Russo and Ottoboni, 2019).

### Clinical Relevance: Moving Concussion Management From Baselines to Normative-Driven Recovery

There have been concerns raised about the validity of baseline testing in general, due in the fact that a baseline test is only a single cross-sectional representation of an individual’s state and might be affected by a variety of internal (e.g., intentional sandbagging) and external factors (Schmidt et al., 2012; Brooks et al., 2016). An inaccurate baseline assessment could thus result in both false positive and false negative diagnoses. In addition, a baseline test operates under the possibly incorrect assumption that each player is fully neurologically healthy. Many contact sports players, particularly at the elite level, have likely sustained prior concussions as well as a history of repetitive head trauma, which can confound baseline assessments. This further highlights the need for objective tests of brain function that are robust, reliable and both sensitive and specific to detecting neurological deviation—independent of baseline testing (Dolan et al., 2007; Smith et al., 2017).

A key question for is how reference data can be applied in a clinical setting to improve longitudinal monitoring for concussion-related effects. The advantage of reference values is an inherent clinical target range to manage toward during rehabilitation from injury, enabling longitudinal monitoring of recovery progress and/or testing efficacy of treatment. Importantly, this approach can incorporate baseline data, where available, but can also be done without it. An example of this application is represented in Figure 3. Presenting data in multivariate radar plot format allows for clinicians to rapidly visualize and identify any deviation from normal, using the average “hexagonal” shape as a quick reference. This feature facilitates a quick and reliable clinical evaluation of functional brain activity that may be compromised after a brain injury. As detailed in Figure 3, the radar plots of a concussed athlete represent a deviation from the “normal” shape that may alert a clinician without the necessity to examine numerical data. Progress in recovery can then be monitored in a longitudinal fashion during patient recovery until the radar plot reaches typical ranges for all variables ahead of any return-to-play decisions. This longitudinal monitoring approach accounts for confounding factors and inherent variability within the individual, but also provides an individual-level statistical framework for evaluating significant change over time. Fickling et al. (2020) demonstrated this clinical application pathway using a longitudinal approach in recovery from severe traumatic brain injury, suggesting similar approaches can be taken for concussion.

The increasing utilization of objective, physiological evaluation in concussion is encouraging, and not just in EEG/ERPs (Corbin-Berrigan et al., 2020). Consensus is emerging that a variety of additional modalities can be useful, such as blood and fluid biomarkers, genetic profiling, and vestibular and oculomotor assessments (Kontos et al., 2017; McCrea et al., 2017; McCrory et al., 2017; Wood et al., 2019; Slavoaca et al., 2020). Recently, machine learning techniques have demonstrated high sensitivity to logistic classification of injury status based on single or multimodal assessments (Jacquin et al., 2018; Boshra et al., 2019; Bazarian et al., 2021). While further validation and integration is required, there is an increasing urgency to develop portable, practical medical technologies based on these scientific findings to better enable point-of-care evaluations (Smith et al., 2017; Yue et al., 2020).

### Caveats

Several caveats to this study should be considered: (1) The sample sizes in this study provided a good start, but continued normative data collection is required. For instance, the current analysis was limited by the age range of the sampled population(s) and further studies to expand the age range are important. Building larger normative databases will also enable further diversification along with more robust conclusions. On-going larger sample data collection continues beyond the scope of this study. Future comparisons will refine and stratify norms, such as closely matched norms for elite athletes in non-contact sports. This is a valuable next step given that, while no prior concussion was reported by any participant, existing subconcussive and/or undisclosed prior injuries may have affected the results (Covassin et al., 2003; Haider et al., 2018; Fickling et al., 2019b, 2021); (2) The inverse problem with specific reference datasets is that they cannot be applied broadly. Indeed, a specific comparison to the general reference databases was selected, in part, to address this issue. Nonetheless, given that the brain vital sign evaluation can be done in under 10-min, it may be advisable to collect specific subsets where possible. In this respect, the current dataset represents the first in the planned aggregation of an open-access large-scale reference value dataset for quantitative EEG (QEEG) (Fickling et al., 2019b); (3) As with all reference comparisons, assumptions cannot be made about an individual’s specific pre-morbid brain vital sign profile, with valuable insight contributed from baseline data where possible. Similar to establishing a profile of hypertensive blood pressure, post-morbid management can often proceed to establish a profile of recovery to reference ranges; (4) While age was modeled as a covariate to best control for differences between groups as a factor of age, a more robust comparison would have been to compare the elite athletes with an appropriately sized sample of age-matched controls. Further increasing the size and diversity of the reference database would better enable these comparisons; and (5) Finally, the specific norms were technically influenced by the situation-specific EEG signal quality at the time of acquisition. Variation in EEG signal quality can have a minor influence the resulting brain vital sign measurements and can therefore introduce some uncontrolled variance during a clinical test. Advances in signal processing and classification have addressed EEG quality assessment and standardization, enabling management of signal quality in the future (Fickling et al., 2019a; Ghosh Hajra et al., 2020).

## Conclusion

The current study reports brain vital sign norms for 58 elite, healthy, male ice-hockey players compared to 135 general control subjects to provide an important clinical alternative to baselining limitations. Reference values for elite, ice-hockey players showed strong predicted test-retest reliability. As expected, there were significant age-related changes across the lifespan, with additional small but significant differences between the elite athlete group and the general reference group after controlling for age as a covariate. The findings support the concept that normative physiological data specific to age, skill level, and experience can be used in concussion evaluation, and combined with general norms and/or individual baseline assessments where appropriate and/or possible. As an objective, physiological measure of cognitive brain function, brain vital sign monitoring can enable both clinical management and performance optimization empirical comparison against a growing open-access reference value dataset.

## Data Availability Statement

The raw data supporting the conclusions of this article will be made available by the authors, without undue reservation. Brain vital signs data generated by this study are available in Supplementary Material.

## Ethics Statement

The studies were reviewed and approved by the institutional review board Advarra. The study design was a cross-sectional observational experiment (Clinicaltrials.gov Identifiers: Group A – NCT03975023, Group B – NCT03835962). Informed, written consent/assent was obtained according to the declaration of Helsinki. Participants over the age of majority provided consent. Participants under the age of majority provided assent, in addition to consent from their parents/guardians.

## Author Contributions

FC, RD’A, GPa, SA, MH, and EO: conceptualization. FC, TF, SF, RD’A, BL, and NC: literature search. FC, RD’A, GPa, SA, MH, EO, NC, BL, and GPw: study design. CS, SF, BL, NC, and GPw: data collection and data verification. CS, SF, and BL: data analysis. SF: the figures. FC, RD’A, SF, BL, and NC: data interpretation. FC, RD’A, SF, BL, NC, and TF: writing – original draft. All authors contributed in writing – review and editing.

## Conflict of Interest

SF, NC, GPw, TF, BL, and RD’A are associated with HealthTech Connex and have a financial interest in the NeuroCatch Platform. FC is the founder of the Carrick Institute, but had no financial interest in the NeuroCatch Platform. The remaining authors declare that the research was conducted in the absence of any commercial or financial relationships that could be construed as a potential conflict of interest.

## Publisher’s Note

All claims expressed in this article are solely those of the authors and do not necessarily represent those of their affiliated organizations, or those of the publisher, the editors and the reviewers. Any product that may be evaluated in this article, or claim that may be made by its manufacturer, is not guaranteed or endorsed by the publisher.
